# Impact of aspirin on bleeding and blood product usage in off‐pump and on‐pump coronary artery bypass graft surgery

**DOI:** 10.1002/jha2.400

**Published:** 2022-02-16

**Authors:** Christopher Little, Zain Odho, Richard Szydlo, Tuan‐Chen Aw, Mike Laffan, Deepa R. J. Arachchillage

**Affiliations:** ^1^ Centre for Haematology, Department of Immunology and Inflammation Imperial College London London UK; ^2^ Department of Biochemistry, Royal Brompton & Harefield Hospitals Part of Guy's & St Thomas’ NHS Foundation Trust London UK; ^3^ Department of Anaesthesia Royal Brompton Hospital & Harefield NHS Foundation Trust London UK; ^4^ Department of Haematology Imperial College Healthcare NHS Trust Imperial College London London UK; ^5^ Department of Haematology Royal Brompton Hospital London UK

**Keywords:** aspirin, bleeding, coronary artery bypass graft, thromboembolism, transfusion

## Abstract

Major bleeding is linked to poorer outcomes following cardiac surgery. Current guidelines recommend continuation of aspirin prior to coronary artery by‐pass graft (CABG) but the effect of continuing aspirin in patients with prior indication for aspirin, in particular during off‐pump CABG (OPCABG), has not been systematically assessed. In this study, we analysed the effect of continuing aspirin prior to OPCABG and on‐pump CABG with respect to bleeding and blood product usage. We compared propensity‐matched cohorts of patients who continued aspirin until the day of OPCABG or CABG to controls (no antiplatelet) and to patients discontinuing aspirin 5–7 days prior. Length of hospital stay, 30‐day mortality and thromboembolism rates were similar for both OPCABG and CABG. During OPCABG, aspirin‐continued patients received more intraoperative red cell units compared to controls without difference in bleeding. Aspirin‐continued patients received more blood products perioperatively and bled more than aspirin‐discontinued patients undergoing OPCABG. The only difference during CABG was a small increase in the volume of cells salvaged among aspirin‐continued patients compared to controls. Current guidelines on the continuation of aspirin prior to CABG and OPCABG are safe. Continuation of aspirin prior to OPCABG may result in more bleeding and blood product usage.

## 1 INTRODUCTION

Cardiovascular disease is a leading cause of morbidity and mortality [1] with platelets playing a major role in the pathogenesis of cardiovascular events [2]. The National Institute for Health and Care Excellence recommends low‐dose (75–100 mg) aspirin monotherapy for angina, peripheral arterial disease, transient ischaemic attack/stroke and after percutaneous coronary intervention following an initial dual‐antiplatelet therapy [3]. Many patients requiring coronary artery by‐pass graft (CABG) are therefore on aspirin.

Major bleeding (> 150 ml of thoracic or mediastinal drainage per hour) is a major complication of cardiothoracic surgery [4] associated with increased blood product usage, intensive treatment unit stay, mortality and cost [5]. Traditionally, aspirin was stopped 5–7 days prior to surgery [6] as antiplatelet therapy is a patient‐related factor that increases risk should inadequate perioperative discontinuation occur [4]. Meta‐analyses however, found no association between bleeding (estimated via chest drain volume) and aspirin doses of < 160 mg taken on the day of CABG [7]. In 2016, a large randomised controlled trial, with the use of tranexamic acid (antifibrinolytic), demonstrated no difference between patients who received aspirin (100 mg) or placebo on the day of CABG in respect of thromboembolic events, re‐operation rates and bleeding [6]. Following this study, it became a standard practice that aspirin be continued prior to cardiac surgeries with discontinuation of therapy reserved for those with high bleeding risk, very low thrombotic risk or refusing transfusion [8]. Neither Myles et al. [6] nor Hastings et al. [7], however, included patients with prior indication for aspirin therapy [6, 7]. Both studies also lacked data on continuing aspirin prior to off‐pump CABG (OPCABG): only 3% of cases in Myles et al. were OPCABG [6] with no OPCABG sub‐group testing by Hastings et al. due to small numbers [7]. Other studies have reported the effect of continuing aspirin until the day of OPCABG, but these too have important limitations including small sample sizes, ambiguous or lack of comparator groups and lack of information on bleeding and blood product use [9, 10, 11, 12].

This study addresses these limitations [9, 10, 11, 12] and better assesses the true in vivo effects of continuing aspirin having eliminated the confounding effects of cardiopulmonary by‐pass (CPB) including high‐dose heparin, loss of large von‐Willebrand Factor (VWF) multimers [13], hypothermia‐induced coagulopathy [14, 15, 16, 17] and protamine‐induced platelet dysfunction [18] via OPCABG analyses. As all included aspirin patients had an indication for therapy prior to surgery, our study population better represents patients taking aspirin prior to CABG as patients included in Myles et al. [6] and Hastings et al. [7] had no history of aspirin use prior to surgery. Comparisons between patients continuing and discontinuing aspirin also enabled analyses of the clinical decision to discontinue aspirin prior to both CABG and OPCABG since the change in British Society of Haematology (BSH) guidelines [8].

The objective of this study was to determine the effect of continuing aspirin on bleeding and blood product use in adult patients (> 18 years) undergoing OPCABG or CABG. We therefore compared propensity‐matched cohorts of patients on long‐term aspirin monotherapy who continued therapy until the day of surgery to controls (not on antiplatelet or anticoagulant) and to patients who discontinued therapy 5–7 days prior, with separate analyses for CABG and OPCABG.

Primary outcome measures were the intraoperative (during surgery), postoperative (end of surgery to 48 h from the start of surgery) and perioperative (48 h from the start of surgery), use of packed red cells (PRC), platelets, cryoprecipitate, fresh frozen plasma (FFP) and salvaged cells; the change in haemoglobin (Hb), packed cell volume (PCV), platelet count following cardiac surgery, the volume of red cells salvaged intraoperatively and the surgical drain volume 24‐h post surgery.

Secondary outcome measures were the initial location of postoperative care, length of hospital stay (LOHS), thromboembolic (TE) rate and 30‐day mortality. TE is defined as objectively confirmed venous or arterial thrombosis within 30 days of the surgery. All patients had clinic review at 6 weeks following the surgery; therefore, even if the thrombosis was diagnosed in another hospital, this information was available.

## 2 PATIENTS AND METHODS

This was a single‐centre, retrospective study in a major tertiary referral centre for cardiothoracic surgery within the United Kingdom. The study was approved by the Research Ethics Committee and the Local Research and Development Office (Reference number: 244283). Data, including any anticoagulant or antiplatelet taken prior to admission and when the antiplatelet was stopped prior to surgery were extracted from the Clinical Data Warehouse and electronic patient records (EPR). Data were cleaned for outliers, and patients with unresolved data quality issues were identified. In both instances, the EPR were checked to correct any errors in the data. Patients with missing data were identified with attempts made to fill in missing data using EPR before exclusion.

### 2.1 Patients

Patients on long‐term aspirin therapy (continued and discontinued 5‐7 days prior) comprised the study groups. Patients not on anticoagulant or with no antiplatelet effect at the time of surgery (either because they had never taken it or had discontinued > 7 days prior) comprised the control group.

### 2.2 Inclusion criteria

Patients ≥ 18 years old undergoing OPCABG or CABG alone from 1 March 2016 to 31 December 2018, with full blood count within 14 days prior to the surgery.

### 2.3 Exclusion criteria

The following patients were excluded: patients on warfarin or direct oral anticoagulants, patients with missing or inaccurate data that could not be retrieved using EPR, patients undergoing procedures other than CABG including patients having further surgery in combination with CABG, patients stopping aspirin 1–4 days prior to surgery, patients on other antiplatelet therapies that discontinued < 7 days of surgery, patients who returned to the theatre following the surgery owing to bleeding that was judged surgical in origin or with no data distinguishing surgical from bleeding related to coagulopathy and patients with any of the following characteristics that independently influence bleeding: cardiac arrest, cardiogenic shock, use of intravenous nitrates, heparin or inotrope, aortic balloon pump insertion, use of a ventricular assist device or any other preoperative support device or thrombolysis within 24 h of surgery.

### 2.4 Statistical analysis

Nearest‐neighbour propensity matching (for demographics and comorbidities) was used to account for differences in baseline patient characteristics. Independent variables used in the propensity matching (Table 1) were patient characteristics that could be assigned on admission and related to the outcomes of interest. Results were presented as percentages, for categorical data; median and range, for skewed continuous data; means (95% confidence interval) when median values equalled 0 to better represent differences. Groups were compared using the Chi‐squared or linear‐by‐linear tests for categorical data, and the *t*‐test, Mann–Whitney test or Kruskal–Wallis test for continuous data (as appropriate). Analyses were performed using SPSS version 24 software (IBM Corp.).

**TABLE 1:** Standard mean differences (SMD) following propensity matching

	**Off‐pump coronary artery by‐pass graft (OPCABG)**		**CABG**	
	**Control versus aspirin‐continued**	**Aspirin‐continued versus discontinued**	**Control versus aspirin‐continued**	**Aspirin‐continued versus discontinued**
**Male sex (%)**	0.0600	0	0.0174	0
**Age (years)**	**0.1696**	0.0019	0.0301	0.0332
**BMI (kg/m^2^)**	**0.2199**	0.0203	0.0262	0.0931
**Previous cardiac surgery (%)**	0.0458	0	0.0418	0
**LVEF > 50% (%)**	0.0179	0.0990	0	0.0268
**Extracardiac arteriopathy (%)**	0.0379	0.0412	0.0499	0.0346
**Neurological dysfunction (%)**	0	0.0700	0.3010	0.0505
**Pulmonary disease (%)**	0.0286	**0.1368**	0.0542	0.0346
**Previous MI (%)**	0	**0.2317**	0	0.0684
**Diabetes (%)**	0.0331	**0.1192**	0.0140	0.0398
**Hypertension (%)**	0.0963	0.0142	0.0335	0
**EuroSCORE 2**	0.0011	0.0672	0.0015	0.0048

*Note*: An SMD < 0.1 was taken to mean a very small effect, < 0.2 a small effect and < 0.5 a moderate effect. Bolds numbers indicate an SMD > 0.1.

Abbreviations: BMI, body mass index; CABG, coronary artery bypass graft; LVEF, left ventricular ejection fraction; MI, myocardial infarction.

## 3 RESULTS

**FIGURE 1:**
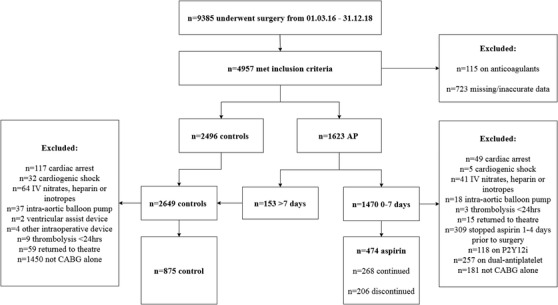
Flow chart of the inclusion and exclusion of the study participants

Figure 1 outlines the inclusion and exclusion criteria with 1349 patients considered for propensity matching. Following propensity matching, the OPCABG study groups comprised 452 patients (122 controls vs. 122 aspirin‐continued and 104 aspirin‐continued vs. 104 aspirin‐discontinued). The CABG study groups included 496 patients (146 controls vs. 146 aspirin‐continued, and 102 aspirin‐continued vs. 102 aspirin‐discontinued). Minor imbalances (standard mean difference > 0.1) were seen between OPCABG cohorts (Table 1), but differences seen in any of these characteristics did not reach significance (Table 2). Both cohorts for CABG analyses were well‐matched (Tables 1 and 2).

**TABLE 2:** Baseline characteristics and interventions in patients who underwent OPCABG

	**Control (*N* = 122)**	**Aspirin‐continued (*N* = 122)**		** Aspirin‐continued (*N* = 104)**	**Aspirin‐discontinued (*N* = 104)**	
Male sex (%)	76.2	78.7	0.646	83.7	83.7	1
Age (years)	67.5 (20–87)	70 (38–85)	0.193	69.5 (38–85)	70 (40–88)	0.996
BMI (kg/m^2^)	27.7 (17.6–45.8)	27.3 (18–48.6)	0.458	27.1 (18–48.6)	27.3 (17.8–39.5)	0.918
Previous cardiac surgery (%)	2.5	3.3	0.701	1	1	1
LVEF > 50% (%)	85.2	82.8	0.892	82.7	85.6	0.772
Extracardiac arteriopathy (%)	4.1	4.9	0.758	4.8	5.8	0.757
Neurological dysfunction (%)	6.6	6.6	1	2.9	1.9	0.651
Pulmonary disease (%)	9.8	9	0.827	9.6	14.4	0.286
Previous MI (%)	36.9	36.9	1	31.7	22.1	0.118
Diabetes (%)	45.1	43.4	0.797	43.3	37.5	0.397
Hypertension (%)	72.1	76.2	0.465	76.9	81.7	0.392
Patient status stable (%)	74.6	68	0.258	67.3	74.0	0.286
EuroSCORE 2	1.4 (0.5–23.2)	1.4 (0.5–17.3)	0.827	1.3 (0.5–17.3)	0.9 (0.5–7.7)	0.022
Hb (g/L)	133 (79–164)	135.5 (72–166)	0.509	136 (72–158)	138 (95–164)	0.151
PCV	0.4 (0.23–0.48)	0.4 (0.21–0.48)	0.575	0.4 (0.21–0.47)	0.41 (0.28–0.49)	0.051
Platelet count (x10^9^)	206.5 (68–466)	187.5 (18–569)	0.067	187 (18–569)	207 (103–432)	0.015
Pre‐op CrCl (ml/min)	87.8 (23.4–214.6)	81.1 (21.1–177.7)	0.238	81.5 (32.1–177.7)	79.8 (30.3–217.9)	0.373
48‐h CrCl mean (ml/min)	87 (14–209)	89.5 (16–272)	0.493	87 (18–272)	96.5 (24–188)	0.346
Pre‐op PT (s)	11.8 (9.7–19.1)	11.8 (9.6–16.4)	0.504	11.8 (10–16.4)	11.7 (9.9–17.9)	0.057
48‐h PT mean (s)	13.5 (10.9–18.1)	15.3 (9.7–25.2)	< 0.001	15.2 (9.7–25.2)	13.8 (11.2–19.3)	< 0.001
Pre‐op APTT (s)	31.3 (26.4–108.8)	33.5 (26–82.8)	0.001	33.9 (26–82.8)	31.3 (24.4–39.1)	< 0.001
48‐h APTT mean (s)	30.1 (24.6–44.4)	31.8 (23.4–47)	< 0.001	31.8 (23.4–47.9)	30.3 (21.8–80.6)	< 0.001
24‐h fibrinogen mean (g/L)	3 (1.2–4.8)	3.2 (1.6–5.5)	0.003	3.2 (1.7–5.5)	2.8 (1.7–4.8)	< 0.001
TXA (%)	71.3	76.2	0.383	78.8	68.3	0.084
APROT (%)	1.6	3.3	0.408	2.9	1	0.313
Operation duration (mins)	240 (80–510)	240 (110–630)	0.674	240 (110–630)	196.5 (100–460)	< 0.001
Elective (%)	77.9	65.6	0.033	64.4	89.4	< 0.001
Lead surgeon consultant (%)	68.9	82.8	0.011	84.6	87.5	0.548

*Note*: Data presented as median (range) and percentages were appropriate.

Abbreviations: APROT, aprotinin; APTT, activated partial thromboplastin time; BMI, body mass index; CrCl, creatine clearance as per Cockcroft–Gault equation; Hb, haemoglobin; LVEF, left ventricular ejection fraction; MI, myocardial infarction; PCV, packed cell volume; PT, prothrombin time; TXA, tranexamic acid.

*p* < 0.05 taken to be significant.

### 3.1 Off‐pump coronary artery bypass graft

Compared to controls, patients who continued aspirin had a lower percentage of elective cases with consultants being the lead surgeon more frequently (Table 2). Patients who continued aspirin also had higher EuroSCOREs and lower platelet counts than controls. Aspirin‐continued patients had higher pre‐op activated partial thromboplastin time (APTT), mean 48‐h prothrombin time (PT) and APTT and 24‐h fibrinogen levels than both controls and aspirin‐discontinued patients (Table 2). Operating times were longer among aspirin‐continued patients with a lower percentage of elective cases in comparison to discontinued patients (Table 2).

#### 3.1.1 Indicators of bleeding

No differences were seen between patients who continued aspirin prior to OPCABG and controls with respect to indicators of bleeding. Patients who continued aspirin bled more than aspirin‐discontinued patients with a greater drop in Hb and PCV, a larger 24‐h drain volume and an increased volume of cells salvaged intraoperatively (Table 3).

**TABLE 3:** Indicators of bleeding in patients who underwent OPCABG

	**Control (*N* = 122)**	**Aspirin‐continued (*N* = 122)**		** Aspirin‐continued (*N* = 104)**	**Aspirin‐discontinued (*N *= 104)**	
** Change Hb**	−32 (−71–26)	−35 (−92–10)	0.207	−**35 (**−**79–10)**	−**30 (**−**73–1)**	**0.013**
**Change PCV**	−0.10 (−0.02–0.1)	−0.11 (−0.03–0)	0.184	−**0.11 (**−**0.02–0)**	−**0.09 (**−**0.02–0)**	**0.01**
**Change Plt count**	−52.5 (−182–76)	−48 (−245–76)	0.97	−48 (−245–73)	−43 (−167–30)	0.173
**24‐h drain volume**	575 (25–1675)	587.5 (25–1925)	0.534	**575 (25–1925)**	**500 (0–1800)**	**0.018**
**Cells salvaged**	1570 (0–4357)	1501 (0–20,044)	0.301	**1501 (0–20,044)**	**0 (0–2947)**	**< 0.001**

*Note*: Data presented as median (range).

Abbreviations: Hb, haemoglobin; PCV, packed cell volume; Plt, platelets.

*p* < 0.05 taken to be significant.

#### 3.1.2 Intraoperative blood product administration

**FIGURE 2:**
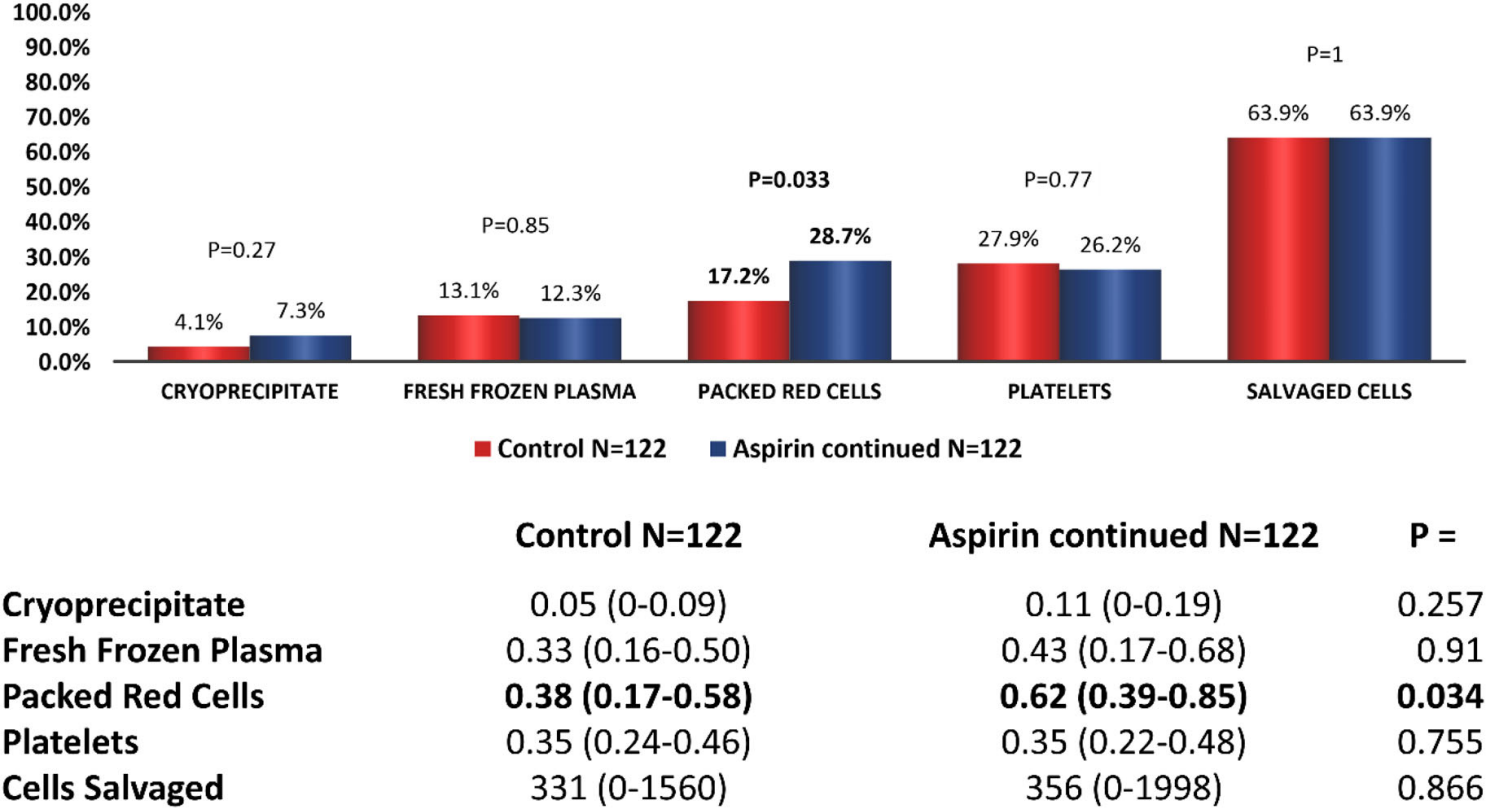
Intraoperative blood product usage in patients who underwent off‐pump coronary artery bypass graft: controls versus aspirin‐continued. The figure represents the percentage of patients in each group receiving each blood product and tables the mean (95% CI) number of units or median (range) volume (ml) of salvaged cells given. *p* < 0.05 taken to be significant

**FIGURE 3:**
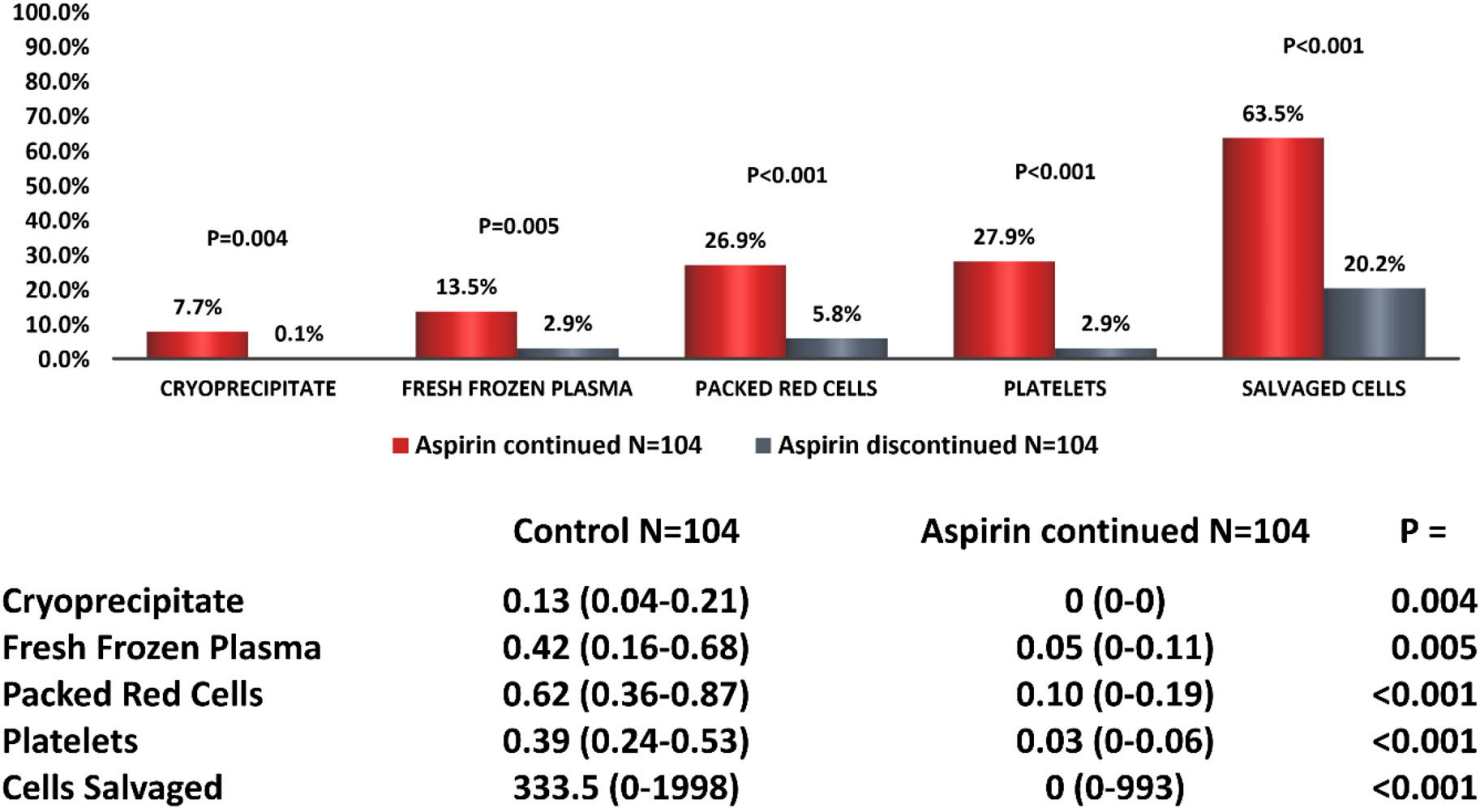
Intraoperative blood product usage in patients who underwent off‐pump coronary artery bypass graft: Aspirin‐continued versus discontinued. The figure represents the percentage of patients in each group receiving each blood product and tables the mean (95% CI) number of units or median (range) volume (ml) of salvaged cells given. *p *< 0.05 taken to be significant

Aspirin‐continued patients received more units of PRC and were more likely to receive PRCs during surgery than controls (Figure 2). Compared to discontinued patients, aspirin‐continued patients received more units of cryoprecipitate, FFP, PRC and platelets and a higher volume of salvaged cells than those discontinuing aspirin intraoperatively. Aspirin‐continued patients were also more likely to receive each product during surgery (Figure 3).

#### 3.1.3 Postoperative blood product administration

There were no differences in postoperative blood product administration for either OPCABG comparison.

#### 3.1.4 Perioperative blood product administration

**FIGURE 4:**
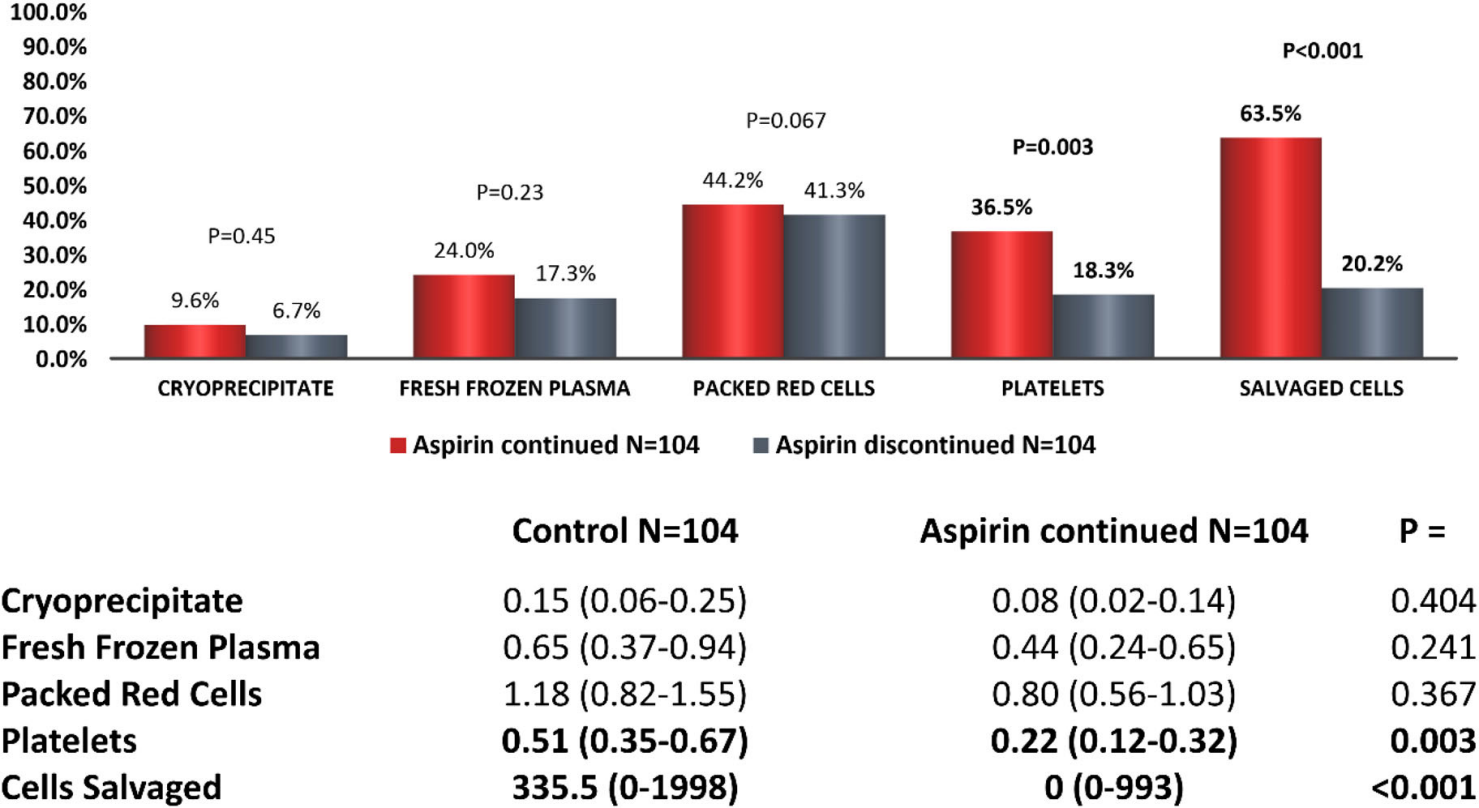
Perioperative blood product usage in patients who underwent off‐pump coronary artery bypass graft: Aspirin‐continued versus discontinued. The figure represents the percentage of patients in each group receiving each blood product and tables the mean (95% CI) number of units or median (range) volume (ml) of salvaged cells given. *p* < 0.05 taken to be significant

Despite differences intraoperatively, there were no overall differences in blood product administration during the perioperative period between controls and those who continued aspirin prior to OPCABG. However, in comparison to discontinued patients, aspirin‐continued patients received more units of platelets and a higher volume of salvaged cells with a higher percentage of patients receiving each of these products (Figure 4).

#### 3.1.5 Postoperative outcomes

There were no differences in the initial location of postoperative care, LOHS, 30‐day mortality or TE rate for either OPCABG comparison (Table 4).

**TABLE 4:** Postoperative outcomes in patients who underwent OPCABG

	**Control (*N* = 122)**	**Aspirin‐continued (*N* = 122)**		** Aspirin‐continued (*N* = 104)**	**Aspirin‐discontinued (*N* = 104)**	
** Location (%)**	75.4 Recovery 2.5 HDU 22.1 ITU	75.4 Recovery 0 HDU 24.6 ITU	0.821	76 Recovery 0 HDU 24 ITU	76.9 Recovery 0 HDU 23.1 ITU	0.87
**LOHS (days)**	9 (5–143)	10 (5–32)	0.789	10 (5–32)	10 (5–90)	0.395
**30‐day mortality (%)**	0.8	2.5	0.313	2.9	1	0.313
**TE event rate (%)**	0.8	0.8	1	0	1	0.316

*Note*: Data presented as percentages and median (range) were appropriate.

Abbreviations: HDU, high dependency unit; ITU, intensive treatment unit; LOHS, length of hospital stay; TE, thromboembolism.

*p* < 0.05 taken to be significant.

### 3.2 Coronary artery bypass graft

#### 3.2.1 Control versus aspirin‐continued

Baseline characteristics following propensity matching for controls and aspirin‐continued patients are summarised in Table S1. Aspirin‐continued patients undergoing CABG had shorter operation durations (*p* = 0.036), a lower percentage of elective cases (*p* = 0.001) and a higher percentage of lead surgeons being of consultant grade (*p* = 0.006) than controls. The pre‐op APTT, mean 48‐h PT (*p* < 0.001) and APTT (*p *= 0.001) and mean 24‐h fibrinogen levels (*p* < 0.001) were also higher in those who continued aspirin. The only difference in bleeding was a small increase in cells salvaged intraoperatively among controls (*p* = 0.01; Table S2). Intraoperative, postoperative and perioperative blood product administration was similar between controls and aspirin‐continued patients. There were no differences in the initial location of postoperative care, LOHS, 30‐day mortality or TE rate between controls and those who continued aspirin prior to CABG (Table S3).

#### 3.2.2 Aspirin‐continued versus discontinued

Table S1 summarises the baseline characteristics of propensity‐matched aspirin‐continued and discontinued patients undergoing CABG. Baseline Hb (*p* = 0.003) and PCV (*p* = 0.005) were marginally but significantly higher in the aspirin‐discontinued cohort. In comparison to discontinued patients, aspirin‐continued patients undergoing CABG had higher pre‐op APTT, mean 48‐h PT and APTT and 24‐h fibrinogen levels (*p* < 0.001 for all). Aspirin‐continued patients also had shorter operation durations (*p *= 0.002) and a lower percentage of elective cases (*p *= 0.001). Aspirin‐discontinued patients bled more than aspirin‐continued patients with a greater drop in Hb (*p* = 0.006), PCV (*p* = 0.005) and platelet count (*p* = 0.015) and a larger volume of cells salvaged intraoperatively (*p* < 0.001; Table S2). During the perioperative period, only the volume of salvaged cells returned differed with a higher volume returned to discontinued patients (*p* = 0.005) who were also more likely to receive salvaged cells (*p* = 0.002; Figure S1). There were no differences in location of postoperative care, LOHS, 30‐day mortality or TE rate (Table S3).

## 4 DISCUSSION

Since the change in aspirin guidelines prior to cardiac surgery in 2016 [8], this is the first study to compare the effects of continuing aspirin prior to OPCABG and CABG to controls and to patients that discontinued aspirin 5–7 days prior to surgery. By analysing OPCABG patients separately, we have addressed limitations of notable studies lacking sufficient OPCABG analyses [6, 7] and better elicit the true in vivo effects of continuing aspirin with respect to bleeding and blood product usage by eliminating the confounding effects of CPB [6, 7, 13–18]. As all included aspirin patients had an indication for monotherapy and through comparisons between aspirin‐continued and discontinued patients, we could also analyse the clinical decision to discontinue aspirin since it became a practice to continue aspirin prior to surgery [8]. Such comparisons will better inform surgeons about the effects of continuing or discontinuing aspirin prior to OPCABG or CABG.

The main finding was the safety of continuing aspirin until the day of OPCABG or CABG as judged by equivalent LOHS, 30‐day mortality and TE rates to both controls and discontinued comparators. Because this is a retrospective study and so unrandomised, it is not possible to match all confounding risk factors. However, these findings suggest that the clinicians’ application of the 2016 criteria for discontinuation (very high bleeding risk, a very low thrombosis risk or declining transfusion) [8] prior to OPCABG or CABG are safe.

An increase in PRC usage intraoperatively during OPCABG was observed among patients who continued aspirin in comparison to controls. However, when looking at only the patients that received blood products, the mean number of units given to each group was similar (2.19 units control vs. 2.17 units aspirin), suggesting a similar intensity of transfusion when required.

Patients who continued aspirin prior to OPCABG also received more products than discontinued patients. This was accompanied by marginal, yet significant increases in bleeding in the continued group. The mean units of cryoprecipitate (1.63 continued vs. 0 discontinued), FFP (3.14 continued vs. 1.67 discontinued), PRC (2.29 continued vs. 1.67 discontinued) and platelets (1.38 continued vs. 1 discontinued) and median volume of salvaged cells (450 ml continued vs. 239 ml discontinued) given in theatre remained higher when analysing only patients receiving blood products. This held true for platelet and cell salvage administration perioperatively. Therefore, when the confounding effect of refusal to consent to transfusion (an indication for discontinuation) is removed, differences in blood product administration still existed.

These findings suggest that when confounding effects of CPB [13, 14, 15, 16, 17, 18] are removed and in vivo effects of aspirin are more noticeable, continuing aspirin prior to OPCABG may increase the requirement for blood products. These differences are more pronounced in comparison to aspirin‐discontinued patients than in comparison to controls given the greater intensity and frequency of blood product usage and increased bleeding not seen in control comparisons. Such findings in OPCABG contradict published works [9, 10, 11, 12], but these studies have significant limitations including: small study sizes (< 25 per group) [9], comparator groups being confounded by patients taking clopidogrel and warfarin within < 7 days of surgery [9], ambiguous group definitions with it being unclear if the comparators were acting more like an aspirin discontinuation group (stopping 5–7 days prior) or a control group (stopping antiplatelet > 7 days prior) [10, 11], failure to assess administration of platelets or cryoprecipitate, intraoperative product administration or changes in haematological parameters following surgery [10], no measurement of mean differences in product administration [11], lack of outcome measures assessing bleeding or blood product usage [12] and lack of comparison to previous guideline discontinuation protocols (stopping 5–7 days prior) [12]. We believe our study includes a more robust assessment of bleeding and blood product usage and better defines comparator groups therefore better describing the effects of continuing aspirin prior to OPCABG. Our comparisons also suggest that clinical decisions to discontinue aspirin were not associated with an increase in thromboembolic events. The small number of events seen, however, means further study is required to better understand the balance between bleeding and TE when deciding to discontinue aspirin prior to OPCABG.

Although aspirin‐continued patients had prolonged mean 48‐h PT and APTT, compared to controls, they were within normal ranges (Table 2). It is therefore unlikely that clotting times impacted the observed results. Not surprisingly, control patients were more likely to undergo an elective OPCABG and were less likely to have a consultant as the lead surgeon. The in vivo effects of these differences are unknown and are a limitation of the retrospective nature of this study. The differences seen between aspirin‐continued and discontinued patients during OPCABG with respect to operation duration and percentage of elective cases may also have confounded results. However, it is possible that the prolonged operation duration was secondary to increased bleeding that, despite increased blood product administration intraoperatively, was still significantly higher in the aspirin‐continued group.

We report no clinically significant differences in bleeding or blood product usage between controls and aspirin‐continued patients undergoing CABG as the difference between the two groups is very small. These results support previous works [6, 7] but add to the literature as we can now suggest that the intensity of blood product transfusion, not just the likelihood of transfusion [6], is similar between controls and those continuing aspirin prior to CABG. We can also now suggest that such conclusions can be applied to patients on long‐term aspirin who continue therapy prior to CABG. As with the OPCABG comparison between controls and aspirin‐continued patients, differences were seen in clotting times, percentage of elective cases and lead surgeon grade despite propensity matching with the same conclusions about the effects of these differences applying here.

Surprisingly, patients who discontinued aspirin prior to CABG bled more than those who continued aspirin. Differences in clotting times were observed but with higher PT and APTT in the aspirin‐continued group, the difference were unlikely to be significant. Operation duration was longer in those discontinuing aspirin prior to CABG (20 min longer), but the percentage of elective cases was higher (87.3% vs. 68.6%). It is possible, however, that the increased operation time may have resulted from the increased bleeding in the discontinuation group. With TE and bleeding risk factors being well‐matched at baseline and similar transfusion rates between groups (refusing of transfusion being a possible indication for discontinuation and a confounding factor), we conclude that other unidentified factors were responsible for the increased bleeding seen.

The main limitations of this study are its retrospective, observational nature and lack of pre‐specified transfusion criteria and power calculations for the outcomes. Clinician bias could therefore not be excluded as an explanation for differences seen although professionals at the centre have great experience of haemostatic practices during high bleeding risk procedures, and so we anticipated minimal bias. However, the bias airing from the decision to continue or discontinue aspirin by the clinician cannot be excluded. Propensity matching analyses were performed to minimise differences in patient cohorts and better facilitate comparison. Whilst these were successful in significantly reducing differences between groups, some differences were still present. These differences in patient characteristics were small and believed to have minimal impact on results; a confounding effect could not be completely excluded.

## 5 CONCLUSION

Current BSH guidelines and practices at the study centre are safe and effective, including the decision to discontinue aspirin 5–7 days prior to OPCABG. However, given low TE rates, this study was likely underpowered to definitively comment on TE. Any increase in blood product administration associated with continuing aspirin monotherapy prior to OPCAB, if required, is small and any increase in bleeding is not associated with adverse outcomes.

## CONFLICT OF INTEREST

The authors declare that there is no conflict of interest.

## AUTHOR CONTRIBUTION

Christopher Little performed the data collection, analysis and writing the first draft of the manuscript. Deepa R. J. Arachchillage was involved in the design of the study, analysing and interpretation of the data and revising the manuscript. Zain Odho performed the propensity matching. Richard Szydlo analysed the data. T. C. Aw collected the data. Mike Laffan interpreted the data and reviewed the manuscript. All authors reviewed and approved the final manuscript.

## Supporting information

Supporting Information
