# Supplementary material for: Impact of aspirin on bleeding and blood product usage in off‐pump and on‐pump coronary artery bypass graft surgery
Source: EJHaem. 2022 Feb 16;3(2):317–25. doi: 10.1002/jha2.400 (PMC9175687; doi:10.1002/jha2.400)
Supplement: Supplementary file 1 — Supporting Information [file JHA2-3-317-s001.docx]

| **Supplementary Table 1 - Baseline Characteristics CABG** | | | | | | |  |
| --- | --- | --- | --- | --- | --- | --- | --- |
|  | **Control**  **N=146** | **Aspirin continued**  **N=146** | **P =** | **Aspirin continued**  **N=102** | **Aspirin discontinued**  **N=102** | **P =** | |
| **Male Sex (%)** | 80.1 | 80.8 | 0.88 | 88.2 | 88.2 | 1 | |
| **Age (years)** | 68 (40-87) | 68 (46-85) | 0.84 | 68 (46-85) | 67 (42-86) | 0.862 | |
| **BMI (kg/m^2^)** | 27.4 (17.7-43.1) | 27.5 (18.1-49.7) | 0.99 | 26.9 (18.1-49.7) | 27.6 (20.5-40.5) | 0.278 | |
| **Previous Cardiac Surgery (%)** | 2.1 | 2.7 | 0.70 | 0 | 0 | 1 | |
| **LVEF >50% (%)** | 77.4 | 74.7 | 0.37 | 83.3 | 84.3 | 0.849 | |
| **Extracardiac Arteriopathy (%)** | 6.8 | 8.2 | 0.66 | 7.8 | 8.8 | 0.8 | |
| **Neurological Dysfunction (%)** | 4.8 | 5.5 | 0.79 | 4.9 | 3.9 | 0.733 | |
| **Pulmonary Disease (%)** | 8.2 | 6.8 | 0.66 | 7.8 | 8.8 | 0.8 | |
| **Previous MI (%)** | 35.6 | 35.6 | 1 | 27.5 | 24.5 | 0.632 | |
| **Diabetes (%)** | 39 | 39.7 | 0.91 | 43.1 | 41.2 | 0.777 | |
| **Hypertension (%)** | 80.1 | 78.8 | 0.77 | 74.5 | 74.5 | 1 | |
| **Patient Status Stable (%)** | 72.6 | 71.9 | 0.90 | 74.5 | 73.5 | 0.873 | |
| **EuroSCORE 2** | 1.36 (0.5-49.2) | 1.32 (0.5-26.3) | 0.86 | 1.2 (0.5-7.6) | 1.02 (0.5-5) | 0.064 | |
| **Hb (g/L)** | 134(80-188) | 132.5 (66-160) | 0.058 | **134 (66-160)** | **139 (98-167)** | **0.003** | |
| **PCV** | 0.4 (0.24-0.55) | 0.39 (0.19-0.48) | 0.098 | **0.4 (0.19-0.47)** | **0.41 (0.29-0.51)** | **0.005** | |
| **Platelet Count (x10^9^)** | 200 (67-377) | 205 (65-404) | 0.73 | 200.5 (80-404) | 206.5 (112-390) | 0.059 | |
| **Pre-op CrCl (ml/min)** | 85 (20.1-182.4) | 85.3 (13-192.1) | 0.57 | 86 (13-192.1) | 85.3 (32.7-162.1) | 0.932 | |
| **48-hour CrCl mean (ml/min)** | 92 (19-254) | 95 (15-228) | 0.66 | 94 (15-228) | 98 (11-257) | 0.265 | |
| **Pre-op PT (s)** | 11.7 (9.6-17.9) | 11.7 (9.4-16.9) | 0.73 | 11.38 (9.4-15.4) | 11.6 (9.8-14.4) | 0.177 | |
| **48-hour PT mean (s)** | **13.9 (10.3-20.4)** | **15.3 (9.7-25.9)** | **<0.001** | **15.1 (9.7-24.8)** | **13.6 (10.4-17.8)** | **<0.001** | |
| **Pre-op APTT (s)** | **30.9 (23.4-62.9)** | **34 (25.9-86.6)** | **<0.001** | **33.5 (25.9-67.4)** | **30.8 (23.3-69.1)** | **<0.001** | |
| **48-hour APTT mean (s)** | **30.5 (21.6-89.5)** | **32.4 (24.8-49.1)** | **0.001** | **32.26 (24.8-48.7)** | **29.3 (16.9-46.2)** | **<0.001** | |
| **24-hour Fibrinogen mean (g/L)** | **2.9 (1.4-6.2)** | **3.7 (1.2-5.5)** | **<0.001** | **3.5 (1.2-5.4)** | **2.9 (1.6-4.9)** | **<0.001** | |
| **TXA (%)** | 77.4 | 78.8 | 0.78 | 79.4 | 82.4 | 0.593 | |
| **APROT (%)** | 4.1 | 1.4 | 0.15 | 0 | 1 | 0.316 | |
| **Operation Duration (mins)** | **257.5 (100-650)** | **240 (85-475)** | **0.036** | **232.5 (96-460)** | **252.5 (150-455)** | **0.002** | |
| **Elective (%)** | **82.2** | **65.1** | **0.001** | **68.6** | **87.3** | **0.001** | |
| **Lead Surgeon Consultant (%)** | **64.4** | **78.8** | **0.006** | 78.4 | 71.6 | 0.258 | |
| Data presented as median (range) and percentages were appropriate. BMI = body mass index. LVEF = left ventricular ejection fraction. MI = myocardial infarction. Hb = haemoglobin. PCV = packed cell volume CrCl = creatine clearance as per Cockcroft-Gault equation. PT = prothrombin time. APTT = activate partial thromboplastin time. TXA = tranexamic acid. APROT = aprotinin. **P<0.05 taken to be significant.** | | | | | | | |

| **Supplementary Table 2 - Indicators of Bleeding CABG** | | | | | | |
| --- | --- | --- | --- | --- | --- | --- |
|  | **Control**  **N=146** | **Aspirin continued**  **N=146** | **P =** | **Aspirin continued**  **N=102** | **Aspirin discontinued**  **N=102** | **P =** |
| **Change Hb** | -36 (-106-8) | -34 (-88-22) | 0.095 | **-33 (-88-12)** | **-40.5 (-79-15)** | **0.006** |
| **Change PCV** | -0.11 (-0.3-0) | -0.1 (-0.3-0.1) | 0.11 | **-0.1 (-0.3-0)** | **-0.12 (-0.2-0)** | **0.005** |
| **Change Plt Count** | -64.5 (-200-136) | -57.5 (-216-57) | 0.58 | **-53.5 (-191-57)** | **-70.5 (-190-19)** | **0.015** |
| **24hr drain volume** | 562.5 (50-2200) | 525 (75-1745) | 0.23 | 575 (75-1745) | 612.5 (100-1975) | 0.161 |
| **Cells salvaged** | **1592.5 (0-6250)** | **1333 (0-5336)** | **0.01** | **1212 (0-3500)** | **1934 (0-4914)** | **<0.001** |
| Data presented as median (range). Hb = haemoglobin. PCV = packed cell volume. Plt = platelets. **P<0.05 taken to be significant.** | | | | | | |

| **Supplementary Table 3 - Postoperative Outcomes CABG** | | | | | | |
| --- | --- | --- | --- | --- | --- | --- |
|  | **Control**  **N=146** | **Aspirin continued**  **N=146** | **P =** | **Aspirin continued**  **N=102** | **Aspirin discontinued**  **N=102** | **P =** |
| **Location (%)** | 80.8 Recovery  0.7 HDU  18.5 ITU | 73.3 Recovery  0 HDU  26.7 ITU | 0.11 | 70.6 Recovery  0 HDU  29.4 ITU | 72.5 Recovery  0 HDU  27.5 ITU | 0.756 |
| **LOHS (days)** | 9 (5-55) | 9 (6-107) | 0.65 | 9 (6-107) | 11 (6-69) | 0.255 |
| **30-day mortality (%)** | 2.7 | 0.7 | 0.18 | 0 | 1 | 0.316 |
| **TE event rate (%)** | 0 | 0.7 | 0.32 | 1 | 2 | 0.561 |
| Data presented as percentages and median (range) were appropriate. LOHS = length of hospital stay. TE = thromboembolism. HDU = high dependency unit. ITU = intensive treatment unit. **P<0.05 taken to be significant.** | | | | | | |

**Supplementary Figure 1:** Perioperative Blood Product Usage CABG Aspirin continued vs discontinued


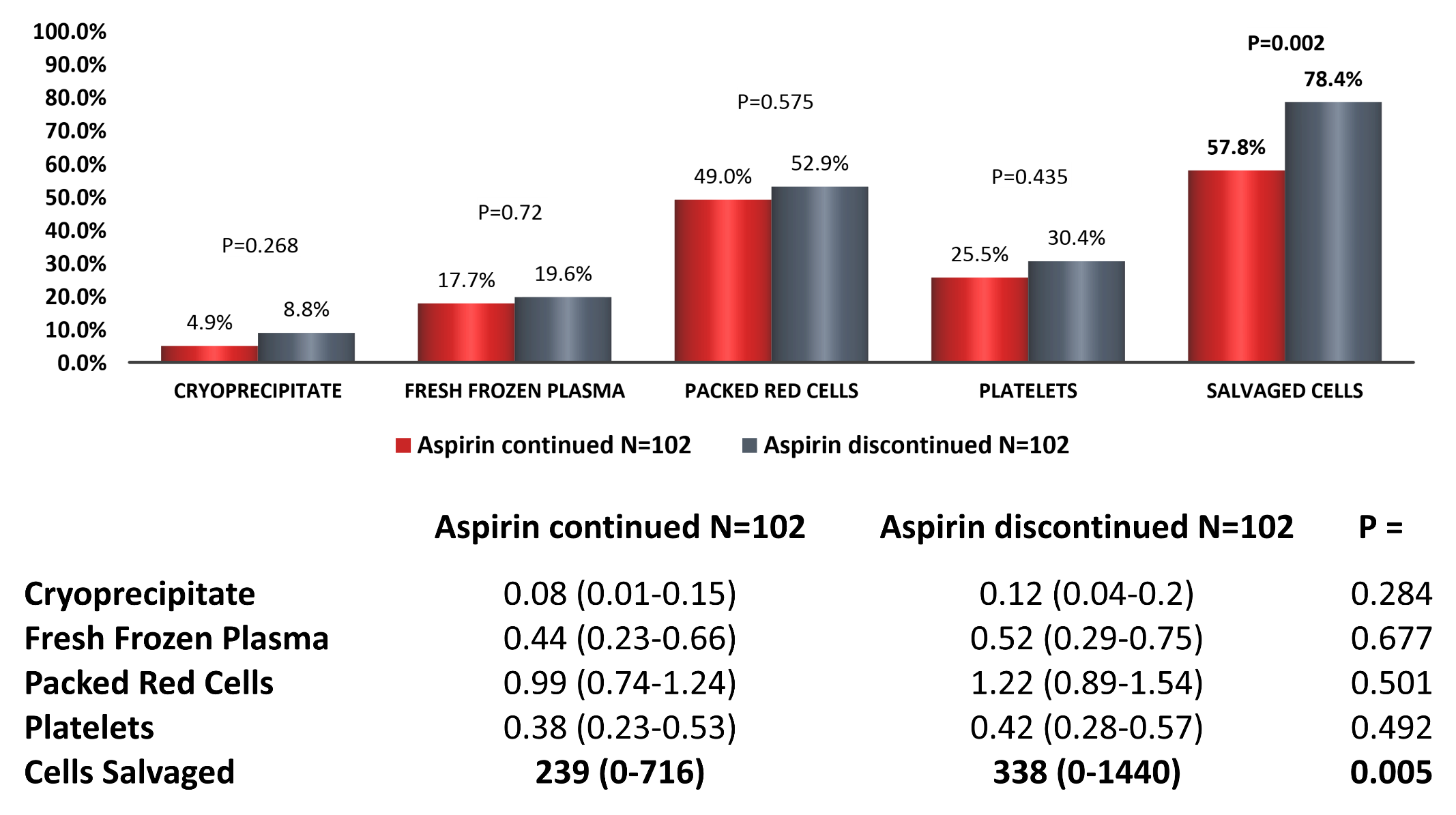


The figure represents the percentage of patients in each group receiving each blood product. The table represents the mean (95% CI) number of units or median (range) volume of salvaged cells given. **P<0.05 taken to be significant.**
